# Radiation Exposure Using Rampart vs Standard Lead Aprons and Shields During Invasive Cardiovascular Procedures

**DOI:** 10.1016/j.jscai.2023.101184

**Published:** 2023-10-19

**Authors:** John C. Lisko, Nikoloz Shekiladze, Joseph Chamoun, Noah Sheikh, Katharine Rainer, Jane Wei, Jose Binongo, Leah Raj, Isida Byku, Stephane Rinfret, Chandan Devireddy, Wissam A. Jaber, Adam B. Greenbaum, Vasilis Babaliaros, Stephen Steuterman, Pratik Sandesara, William J. Nicholson

**Affiliations:** a Section of Interventional Cardiology, Emory University School of Medicine, Atlanta, Georgia; b Emory Structural Heart and Valve Center, Emory University School of Medicine, Atlanta, Georgia; c Department of Biostatistics and Bioinformatics, Rollins School of Public Health, Emory University, Atlanta, Georgia; d Medical Physics Solutions, Inc, San Diego, California

**Keywords:** invasive cardiology, left heart catheterization, radiation safety

## Abstract

**Background:**

Radiation exposure during invasive cardiovascular procedures remains an important health care issue. Lead aprons and shields (LAS) are used to decrease radiation exposure but leave large portions of the body unshielded. The Rampart IC M1128 is a portable radiation shielding system that may significantly attenuate radiation exposure.

**Methods:**

Catheterization laboratory teams were randomized in a 1:1 fashion to perform elective invasive cardiovascular procedures utilizing either traditional LAS or the Rampart IC M1128. Radiation exposure was measured using real-time dosimetry monitoring in prespecified anatomic locations on 3 operators (position 1: first operator/fellow; position 2: second operator/attending; and position 3: catheterization laboratory nurse/technologist). Radiation exposure was measured on a per-case basis.

**Results:**

In total, 100 consecutive cases were randomized in this study (47 Rampart; 53 LAS). There was no difference in fluoroscopy time (12.3 minutes for Rampart vs 15.4 minutes for LAS; *P* = .52), dose area product (288 Gy⋅cm^2^ for Rampart vs 376.5 Gy⋅cm^2^ for LAS; *P* = .52), or scatter radiation (38.8 mRem for Rampart vs 46.8 mRem for LAS; *P* = .61) between the groups. There was significantly lower total body radiation (in milliroentgen equivalent man) exposure using the Rampart than that using LAS for each team member: position 1—0.1 mRem for Rampart vs 2.2 mRem for LAS; *P* < .001; position 2—0.1 mRem Rampart vs 3.2 mRem LAS; *P* < .001; and position 3—0.0 mRem for Rampart vs 0.8 mRem for LAS; *P* < .001.

**Conclusions:**

During routine clinical procedures, the Rampart system significantly decreases total body radiation exposure compared with traditional LAS.

## Highlights

• Rampart reduces total body radiation compared to traditional lead aprons and shields.
• Rampart significantly reduces radiation to the head and neck.
• Rampart enables lead-free invasive cardiac procedures.

## Central Illustration

**Figure:**
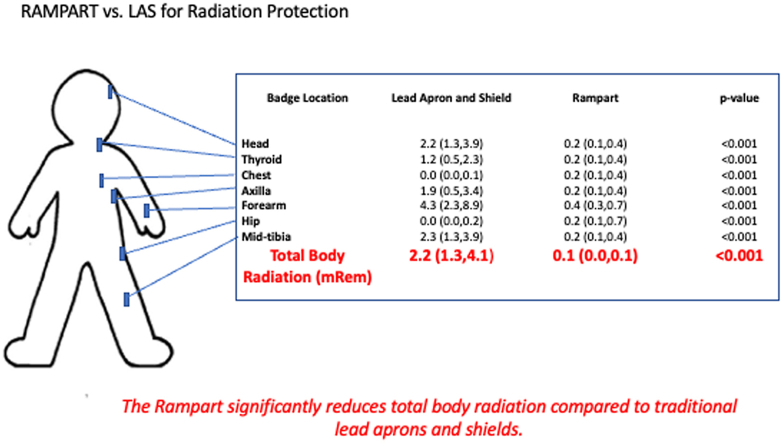

## Introduction

Chronic radiation exposure and orthopedic injuries are well-described occupational hazards to cardiac catheterization laboratory teams.^1^, ^2^, ^3^, ^4^, ^5^, ^6^ Wearable lead aprons and suspended lead shields (lead aprons and shields [LAS]) are the standard approach to mitigate radiation exposure in most invasive cardiovascular procedures.^7^ While effective, these devices leave significant areas of the body exposed and may cause significant musculoskeletal strain^8^—a point well demonstrated by the incidence of left-sided brain tumors in interventional cardiologists^9^ and higher incidence of musculoskeletal pain^5^ in employees exposed to radiation compared to other similar health care workers.^4^

Current imaging systems have significantly decreased radiation exposure to patients, but there has been little innovation in the development of protective equipment for the catheterization laboratory team. An ideal device would effectively protect the entire team from ionizing radiation,^10^ be ergonomically favorable in all invasive cardiovascular procedures, and be portable.

We aimed to compare radiation reduction between the Rampart M1128 (Rampart) portable shielding system versus wearable lead aprons and suspended lead shields during invasive cardiovascular procedures. Herein, we present the results of 100 randomized cases.

## Methods

### Study design

**Figure 1:**
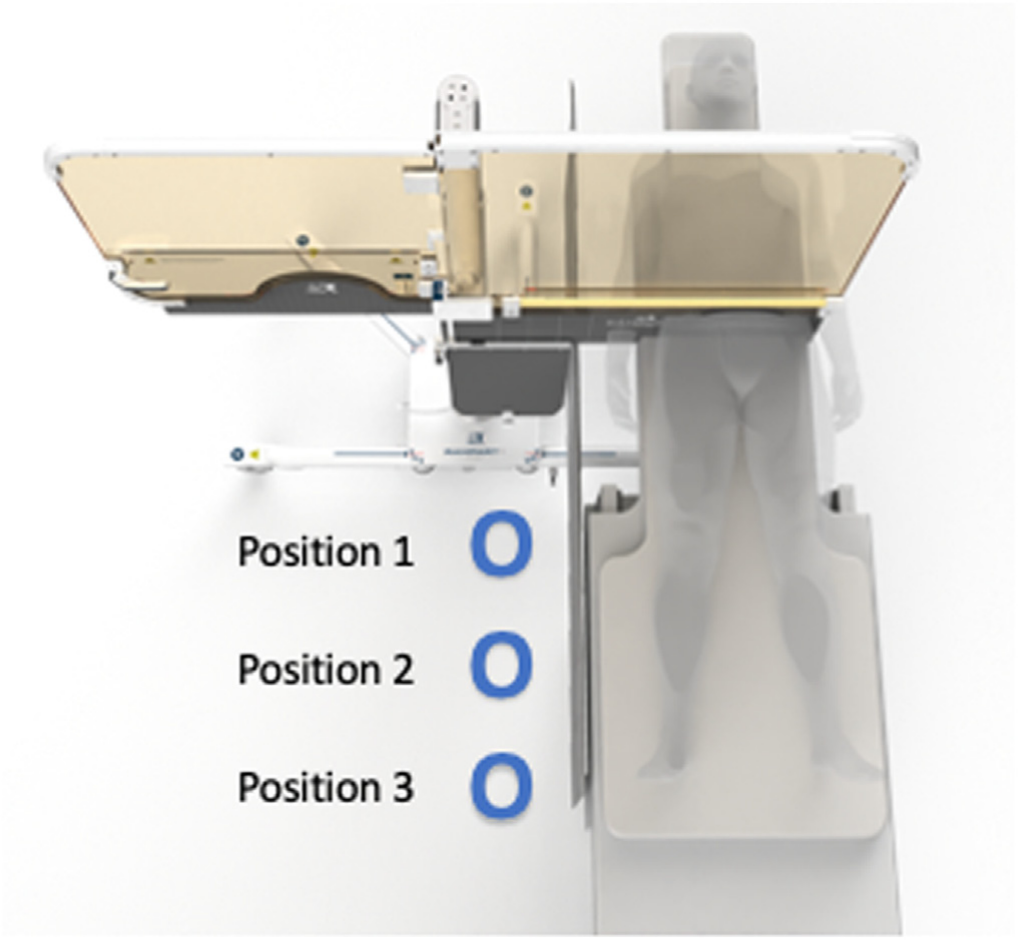
**Rampart M1128: device design.** The Rampart M1128 is a fully adjustable, configurable, floor-supported portable shielding system. The panels are 1.0-mm-thick lead equivalent. A sterile plastic drape is placed over the device for invasive procedures.

This is a single-center, prospective, randomized study of 100 consecutive elective invasive cardiac procedures in which 3 members of the catheterization laboratory team were randomized in a 1:1 fashion, using an internet-based simple randomization design, to wear traditional LAS or use the Rampart M1128 without LAS. The number of cases was selected to complete the study in a timely fashion. Positions 1 and 2 were physicians, and position 3 was a nurse or technologist (Figure 1). There were 2 physicians in each case as the study was conducted at an academic training site with fellow involvement. The study was conceived, designed, and conducted by investigators at Emory Healthcare. The Emory University institutional review board approved the protocol, and all participants provided informed consent.

**Central Illustration:**
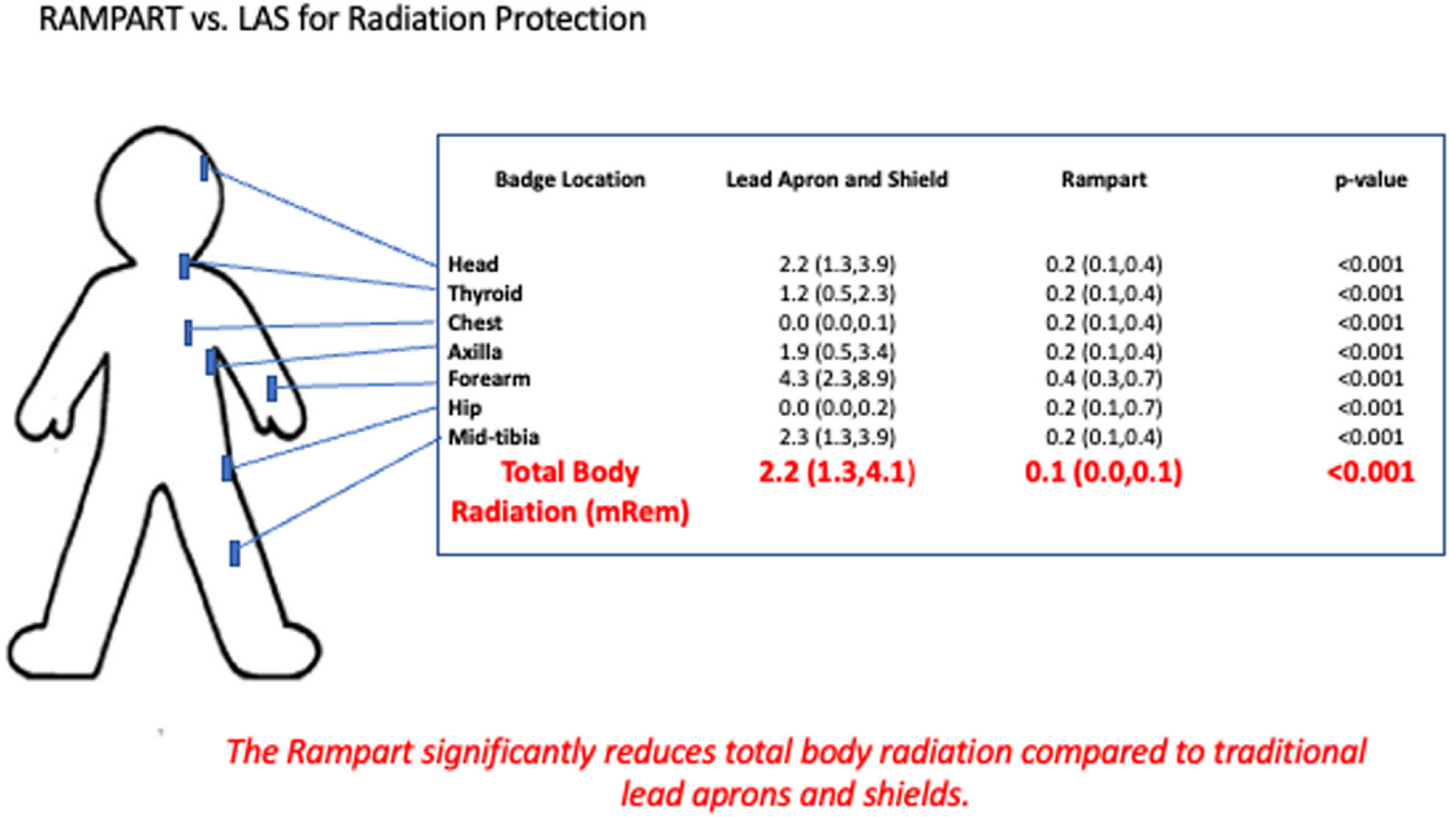
Radiation reduction. A representative example of radiation exposure the first operator. The Rampart M1128 significantly decreases total body radiation, most notably to areas not fully protected by wearable lead aprons and shields (LAS).

Data were prospectively collected in 2 cardiac catheterization laboratories utilizing a Phillips Azurion Clarity IQ fluoroscopy system. All elective coronary and structural heart procedures were eligible for enrollment in the study. Emergency and salvage procedures (eg, ST segment–elevated myocardial infarction) were not eligible for enrollment. Analyses were performed according to the intention-to-treat principle; no patient was switched from one group to the other.

### Radiation shielding

Rampart M1128 is a fully adjustable, configurable, floor-supported, portable shielding system with recommended 2-piece under-table lead and an abdominal protector. The system is a commercial stock–approved device. The portable shielding system has casters on the floor to allow for movement and is placed on the right side of the table (patient right). It has 2 panels (each of 1.0-mm-thick lead equivalent), a center mast, and 2 soft shielding on each panel (0.5-mm-thick lead). The system works in conjunction with table-mounted shields and replaces the need for a ceiling-suspended shield. A sterile plastic drape that requires approximately 2 minutes to place is used per procedure (Figure 1).

For cases utilizing LAS, staff members wore 0.5- to 1-mm-thick standard lead aprons that consisted of a thyroid collar, vest, and skirt. The choice of full-wrap or frontal-wrap apron was at the discretion of the individual. The position of the table and ceiling-mounted lead shields were at the discretion of the operating physician in keeping with standard practice. A RADPAD (Worldwide Innovations and Technologies) was not used in either LAS or Rampart cases.

### Radiation monitoring

The RaySafe i3 (GE Healthcare) is a real-time, wearable, dosimeter with a detection limit of <30 μSv/h, with a dose reproducibility of 10% or 1 μSv. A dosimeter was placed under the fluoroscopy table at a standard distance from position 1 to determine scatter radiation. During the study, each physician and staff member wore multiple RaySafe i3 dosimeters at prespecified locations. Dosimeter locations for positions 1 and 2 were as follows:

1. Head
2. Thyroid
3. Chest
4. Under chest lead (an additional badge was placed under lead for cases randomized to LAS)
5. Axilla
6. Forearm
7. Left hip (below lead)
8. Midtibia

Position 3 did not wear a chest (below lead) or midtibial dosimeter. Radiation exposure was extracted from the devices and stored on a secure computer. Participants were blinded to their radiation exposure during and after each case.

### Total body radiation exposure

The National Council on Radiation Protection and Measurements provides correction factors for dosimeters worn with lead aprons to estimate a whole-body radiation dose. In keeping with standard practice of our institution, we applied the standard EDE2 calculation for cases using LAS, expressed as follows: assigned deep dose equivalent = 0.3 (collar deep dose equivalent). Because the Rampart system serves as “external lead,” the EDE2 calculation is not applicable. In consultation with a medical physicist, total body dose was calculated by averaging the dose recorded by each RaySafe i3 device.

### Data analysis

Quantitative baseline characteristics and radiation readings were summarized as median (lower quartile, upper quartile) or mean (SD) as appropriate. Groups were compared using the Mann-Whitney *U* test or *t* test, as appropriate. All tests of hypothesis were 2-sided, and a .05 level of significance was used. There was no adjustment for multiple comparisons; furthermore, as the same group of operators performed procedures using equal fluoroscopic systems; the operator effect and the catheterization laboratory effect were deemed negligible and therefore not taken into account in the statistical analyses. All analyses were performed with SAS, version 9.4 (SAS Institute).

## Results

### Case description

In total, 100 elective invasive cardiovascular procedures met the inclusion criteria and were randomized in this study. Procedural characteristics are described in Table 1. The Rampart system was successfully positioned in all cases. The Rampart did not limit fluoroscopic projections. Vascular access was obtained following placement of the Rampart system. Most cases in both groups were left heart catheterizations (70% LAS, 55% Rampart) and utilized radial access (45% LAS, 47% Rampart). Structural heart procedures included transcatheter aortic valve replacement, transcatheter mitral valve repair/replacement, transcatheter tricuspid valve repair, and percutaneous septal myotomy. Median total fluoroscopy time was 15.4 (7.3, 30.6) minutes using LAS vs 12.3 (5.1, 28.6) minutes using Rampart (*P* = .52). Control scatter radiation measurements were similar between the groups (46.8 [20.0, 124.0] mRem for LAS vs 38.8 [19.0, 69.9] mRem for Rampart; *P* = .61). There was no statistically significant difference in the dose area product between groups (376.5 [226.0, 1037.0] Gy⋅cm^2^ for LAS vs 288.0 [123.0, 531.0] Gy⋅cm^2^ for Rampart; *P* = .52). There was no difference in the body surface area of patients in the LAS cohort (mean, 2.1 ± 0.42 m^2^) vs Rampart cohort (mean, 2.0 ± 0.27 m^2^; *P* = .1).

**Table 1:** Procedural characteristics.

	Lead apron and shield (n = 53)	Rampart (n = 47)	*P*
Fluoroscopy time, min	15.4 (7.3, 30.6)	12.3 (5.1, 28.6)	.52
Dose area product, Gy⋅cm^2^	376.5 (226.0, 1037.0)	288.0 (123.0, 531.0)	.52
Control reading, mRem	46.8 (20.0, 124.0)	38.8 (19.0, 69.9)	.61
Procedure			
Left heart catheterization ± intervention	37 (70)	26 (55)	–
Structural intervention	7 (13)	13 (28)	–
Right and left heart	4 (7)	4 (9)	–
Catheterization			
Renal angiography ± intervention	2 (4)	2 (4)	–
Right heart catheterization	1 (2)	1 (2)	–
Pulmonary angiography	0	1 (2)	–
Not specified	2 (4)	–	–
Vascular access			
Radial	24 (45)	22 (47)	–
Femoral	20 (38)	14 (30)	–
Radial + femoral	7 (13)	9 (19)	–
Not specified	2 (4)	2 (4)	–

Values are median (first quartile, third quartile) or n (%).

### Radiation exposure to position 1

While using LAS and Rampart system, the operator at position 1 recorded a median total body radiation dose of 2.2 (1.3, 4.1) mRem and of 0.1 (0.0, 0.1) mRem, respectively (Table 2; Central Illustration). Compared to LAS, the Rampart reduced total body radiation by 95%. Table 3 demonstrates radiation to each prespecified measurement location for position 1.

**Table 2:** Total body radiation exposure.

Operator position	Lead apron and shield (n = 53)	Rampart (n = 47)	*P*
Position 1	2.2 (1.3, 4.1)	0.1 (0.0, 0.1)	<.001
Position 2	3.2 (2.0, 4.6)	0.1 (0.0, 0.1)	<.001
Position 3	0.8 (0.4, 1.3)	0.0 (0.0, 0.1)	<.001

Radiation exposure was measured in milliroentgen equivalent man (mRem), summarized using median (first quartile, third quartile). Difference in radiation exposure was assessed using the Mann–Whitney *U* test.

**Table 3:** Radiation exposure to position 1.

Badge location	Lead apron and shield (n = 53)	Rampart (n = 47)	*P*
Head	2.2 (1.3, 3.9)	0.2 (0.1, 0.4)	<.001
Thyroid above lead	1.2 (0.5, 2.3)	0.2 (0.1, 0.4)	<.001
Chest above lead	1.5 (0.7, 3.9)	0.2 (0.1, 0.4)	<.001
Chest below lead	0.0 (0.0, 0.1)	0.2 (0.1, 0.4)	<.001
Axilla	1.9 (0.5, 3.4)	0.2 (0.1, 0.4)	<.001
Forearm	4.3 (2.3, 8.9)	0.4 (0.3, 0.7)	<.001
Hip below lead	0.0 (0.0, 0.2)	0.2 (0.1, 0.7)	<.001
Midtibia	2.3 (1.3, 3.9)	0.2 (0.1, 0.4)	<.001

Radiation exposure was measured in milliroentgen equivalent man (mRem), summarized using median (first quartile, third quartile). Difference in radiation exposure was assessed using the Mann–Whitney *U* test.

### Radiation exposure to position 2

While using LAS and the Rampart system, the operator at position 2 experienced a median total body radiation dose of 3.2 (2.0, 4.6) mRem and of 0.1 (0.0, 0.1) mRem, respectively. Compared to LAS, the Rampart reduced total body radiation by 97%. Table 4 demonstrates radiation to each prespecified measurement location for position 2.

**Table 4:** Radiation exposure to position 2.

Badge location	Lead apron and shield (n = 53)	Rampart (n = 47)	*P*
Head	1.7 (1.0, 2.5)	0.1 (0.1, 0.3)	<.001
Thyroid above lead	1.7 (1.0, 2.7)	0.2 (0.1, 0.4)	<.001
Chest above lead	2.8 (1.3, 4.9)	0.3 (0.1, 0.4)	<.001
Chest below lead	0.1 (0.0, 0.1)	0.3 (0.1, 0.4)	<.001
Axilla	2.6 (1.8, 4.9)	0.2 (0.1, 0.4)	<.001
Forearm	5.5 (2.9, 11.1)	0.4 (0.1, 0.6)	<.001
Hip below lead	0.1 (0.0, 0.4)	0.1 (0.0, 0.3)	.93
Midtibia	2.9 (1.8, 9.0)	0.2 (0.1, 0.4)	<.001

Radiation exposure was measured in milliroentgen equivalent man (mRem), summarized using median (first quartile, third quartile). Difference in radiation exposure was assessed using the Mann–Whitney *U* test.

### Radiation exposure to position 3

While using LAS and the Rampart system, the operator at position 3 recorded a median total body radiation dose of 0.8 (0.4, 1.3) mRem and of 0.0 (0.0, 0.1) mRem, respectively. Compared to LAS, the Rampart reduced total body radiation by nearly 100%. Table 5 demonstrates radiation to each prespecified measurement location for position 3.

**Table 5:** Radiation exposure to position 3.

Badge location	Lead apron and shield (n = 53)	Rampart (n = 47)	*P*
Head	1.0 (0.5, 1.4)	0.1 (0.0, 0.2)	<.001
Thyroid above lead	0.5 (0.3, 1.2)	0.1 (0.0, 0.2)	<.001
Chest above lead	0.5 (0.3, 1.0)	0.1 (0.0, 0.2)	<.001
Axilla	0.6 (0.3, 1.0)	0.0 (0.0, 0.1)	<.001
Forearm	0.9 (0.6, 1.5)	0.2 (0.1, 0.3)	<.001
Hip below lead	0.0 (0.0, 0.0)	0.1 (0.0, 0.2)	.89

Radiation exposure was measured in milliroentgen equivalent man (mRem), summarized using median (first quartile, third quartile). Difference in radiation exposure was assessed using the Mann–Whitney *U* test.

### Radiation exposure to the chest under lead and hip

The median radiation exposure to the chest under lead for cases randomized to LAS was 0.0 (0.0, 0.1) mRem and 0.1 (0.0, 0.1) mRem for position 1 and position 2, respectively. This was a statistically but not clinically significant lower amount of radiation to the chest than that with Rampart: 0.2 (0.1, 0.4) mRem and 0.3 (0.1, 0.4) mRem for position 1 and position 2, respectively. Similarly, there was a statistically higher but clinically insignificant increase in radiation exposure to the hip of operator at position 1 for cases randomized to Rampart (0.2 [0.1, 0.7] mRem vs 0.0 [0.0, 0.2] mRem for LAS; *P* < .001).^7^ There was no difference in hip radiation exposure for position 2 (0.0 [0.0, 0.2] mRem for Rampart vs 0.1 [0.0, 0.3] mRem for LAS; *P* = .93) and position 3 (0.1 [0.0, 0.2] mRem for Rampart vs 0.0 [0.0, 0.0] mRem for LAS; *P* = .89).

### Radiation exposure in areas not covered by LAS

The Rampart significantly reduced radiation exposure to the head, axilla, forearm, and midtibia compared to LAS. Catherization laboratory team members received 10-17 times less radiation to the head, 6-13 times less radiation to the axilla, 11-14 times less radiation to the forearm, and 12-15 times less radiation to the midtibia based on position.

## Discussion

In the present study, we prospectively investigated the feasibility and efficacy of the Rampart M118 portable radiation shielding system to attenuate radiation exposure to the entire cardiac catheterization laboratory team during elective invasive cardiac procedures compared to those of traditional LAS. To our knowledge, this is the first randomized study of a commercially available radiation shielding device. The key findings from the study are as follows: (1) using the Rampart M118 system is technically feasible in coronary and structural heart invasive cardiac procedures; (2) the Rampart system significantly decreases total body radiation compared to LAS; (3) the Rampart system significantly decreases radiation exposure to body areas not traditionally protected by lead aprons and shields; and (4) unlike other novel radiation shielding systems, the Rampart provides significant radiation attenuation to all members of the procedural team.

Our findings have significant implications for the practice of invasive vascular procedures requiring ionizing radiation. Importantly, a diverse case mix was included in this study from left heart catheterizations to first-in-man structural heart interventions. Despite the physical barrier created by the Rampart system, there were no limitations in the operator’s ability to obtain vascular access, obtain required fluoroscopic projections, or perform the procedure. There were no complications caused by the Rampart system or limitations in treating procedural complications with the system in place. While not included in this study, the Rampart M1128 is routinely used during electrophysiologic, endovascular, and neurointerventional procedures.

This study directly and comprehensively measured radiation exposure to prespecified anatomic areas. For each position, the Rampart decreases total body radiation exposure by ≥95% compared to LAS. As expected, the Rampart system significantly reduced radiation exposure to areas not traditionally covered by lead aprons. These effects are most pronounced in the 11-fold reduction in radiation exposure to the head and axilla. Given the known increased risk of brain malignancy,^9^ cataracts,^11^ and carotid atherosclerosis,^12^ these findings are of special significance to practicing invasive cardiologists. This effect is likely magnified in complex, time-intensive procedures.

The Rampart M1128 significantly attenuates radiation to allow for high-volume, complex, invasive cardiovascular procedures. Based on these data, a physician who remained solely in position 1 could perform a 1-Gy case 14,400 times before reaching the yearly dose limit of 5 Rem using Rampart compared to 855 times using LAS. This finding may be of more significance to nonphysician members of the catheterization laboratory team who are often involved in more cases per day than any 1 physician.

The Rampart provides a physical barrier to radiation without adding the weight of lead aprons to any provider. While not studied in this analysis, the Rampart should theoretically decrease orthopedic injury and chronic pain. This has major implications for the field with cardiologists reporting a higher incidence multilevel disc disease and missed worked days compared to other physicians.^6^

Radiation exposure has been consistently cited as a reason for a lack of women in the field of invasive cardiology. The significant radiation reduction provided by the Rampart system may prove to be a useful tool in increased representation of women in the field of cardiology—especially trainees and staff who are often women of childbearing age.^13^, ^14^, ^15^

Multiple radiation shielding systems are commercially available. Zero-Gravity (Biotronik) utilizes a 1.0-mm suspended lead body shield that magnetically engages with a vest worn by the primary operator, attached to a 0.5-mm lead acrylic face shield. Compared to LAS, the device was associated with a significant reduction in head-level physician radiation doses.^16^^,^^17^ Unlike Rampart, Zero-Gravity has not been studied in a prospective, randomized fashion and does not decrease radiation exposure to nonphysician members of the catheterization laboratory team. The Cathpax AIR cabin (Lemer Pax) is a mobile radiation protection cabin that provides 1.0-2.0 mm of lead equivalence to operators during fluoroscopic procedures. The feasibility of the system was demonstrated in a prospective, nonrandomized, all-comers study of 119 consecutive invasive cardiac procedures with a first-operator relative radiation exposure reduction of 78% at the chest and 70% at the wrist.^18^ The study did not examine total body radiation exposure and radiation exposure to nonphysician members of the catheterization laboratory team. The Protego system is a compact, nonportable, radiation shielding system that has been evaluated in a preclinical study with a C-arm position of 40° left anterior oblique. Dose reductions at the location of the primary operator ranged from 97.8% to 99.8% with a >94.2% reduction in scatter radiation at all reference points.^3^ Unlike the Rampart, the device is not fully portable and has not been studied in a prospective, randomized, fashion.

The Rampart system is not designed to attenuate radiation to the left side of the catheterization laboratory. There is an unmet need for a similar system to protect staff, especially anesthesiologists, interventional imagers, and sonographers who work exclusively to the left side of the patient.

### Limitations

Several limitations merit attention in our study. This is a single-center study that did not account for heterogeneity in fluoroscopy systems and case type. However, this study included a diverse case mix with similar fluoroscopic times, dose area products, and similar scatter radiation. Additional radiation safety training was not provided prior to initiation of the study. While this may have decreased overall radiation doses, we feel that our study is reflective of real-world practice. The location of procedures is determined at the discretion of the catheterization laboratory management team. Because of this, there were more coronary cases were randomized than structural heart procedures. Similar fluoroscopic systems were used for all procedures, and we do not believe that there was a significant catheterization laboratory effect. The Rampart was successfully used during multiple structural heart interventions such as transcatheter aortic valve replacement, research transcatheter mitral valve repair/replacements, and a first-in-man percutaneous electrosurgical myectomy. Additionally, tube angulation, frame rates, and field size were not recorded possibly introducing residual confounders in our analysis. There may be variability in operator’s use of optimal radiation safety practices such as collimating filters and opposing the ceiling-suspended shields. While this may introduce bias into the study, the same group of operators performed cases using LAS and Rampart, and we do not believe this significantly skews the outcome. As this study was conducted at a training center with fellow involvement, procedure and fluoroscopic times may have been increased compared to those in routine practice; however, this would not be imbalanced between the Rampart and LAS groups. Similarly, operators may change position during the cases; however, we do not believe that this would significantly impact overall radiation exposure and is unlikely to be systematically biased between cases. While there was no crossover in the study, there was a slight deviation from 1:1 randomization inherent to the simple randomization design. As this was a small imbalance we do not feel that it significantly impacted the outcome of the study and therefore no statistical adjustment was performed in the analysis. Complete patient data were not collected for the study as catheterization laboratory teams and not patients were consented and randomized. The RADPAD is a radiation protection device that is placed on the patient’s body and minimizes the amount of radiation exposure. The device was not used during our study and may have the potential to further decrease radiation scatter. The Rampart system does not provide radiation protection for left-sided procedures and still requires individuals working on the patient’s left side to wear lead. Additionally, the Rampart must be positioned correctly to provide radiation attenuation as reported in our analysis. During the installation process, the manufacturer provides detailed training and proctoring to correctly use the device.

## Conclusions

The Rampart M1128 significantly decreases total body radiation exposure to the catheterization laboratory team during invasive cardiovascular procedures compared to the use of traditional LAS. The results of this study have significant implications for the health and occupational safety of catheterization laboratory teams. The Rampart M1128 may safely enable invasive cardiovascular procedures without the need to wear lead.
